# Deep Learning Prediction of Axillary Lymph Node Metastasis in Breast Cancer Patients Using Clinical Implication-Applied Preprocessed CT Images

**DOI:** 10.3390/curroncol31040169

**Published:** 2024-04-18

**Authors:** Tae Yong Park, Lyo Min Kwon, Jini Hyeon, Bum-Joo Cho, Bum Jun Kim

**Affiliations:** 1 Medical Artificial Intelligence Center, Doheon Institute for Digital Innovation in Medicine, Hallym Univesity Medical Center, Anyang-si 14068, Republic of Korea; taeyong@mach.hallym.or.kr; 2 Department of Radiology, Hallym University Sacred Heart Hospital, Hallym University College of Medicine, Anyang-si 14068, Republic of Korea; lyominkwon@hallym.or.kr; 3 School of Medicine, Hallym University College of Medicine, Chuncheon 24252, Republic of Korea; hji9432@naver.com; 4 Department of Ophthalmology, Hallym University Sacred Heart Hospital, Hallym University College of Medicine, Anyang-si 14068, Republic of Korea; 5 Division of Hematology-Oncology, Department of Internal Medicine, Hallym University Sacred Heart Hospital, Hallym University College of Medicine, Anyang-si 14068, Republic of Korea

**Keywords:** deep learning, convolutional neural network, axillary lymph node metastasis, computed tomography, breast cancer

## Abstract

**Background:** Accurate detection of axillary lymph node (ALN) metastases in breast cancer is crucial for clinical staging and treatment planning. This study aims to develop a deep learning model using clinical implication-applied preprocessed computed tomography (CT) images to enhance the prediction of ALN metastasis in breast cancer patients. **Methods:** A total of 1128 axial CT images of ALN (538 malignant and 590 benign lymph nodes) were collected from 523 breast cancer patients who underwent preoperative CT scans between January 2012 and July 2022 at Hallym University Medical Center. To develop an optimal deep learning model for distinguishing metastatic ALN from benign ALN, a CT image preprocessing protocol with clinical implications and two different cropping methods (fixed size crop [FSC] method and adjustable square crop [ASC] method) were employed. The images were analyzed using three different convolutional neural network (CNN) architectures (ResNet, DenseNet, and EfficientNet). Ensemble methods involving and combining the selection of the two best-performing CNN architectures from each cropping method were applied to generate the final result. **Results:** For the two different cropping methods, DenseNet consistently outperformed ResNet and EfficientNet. The area under the receiver operating characteristic curve (AUROC) for DenseNet, using the FSC and ASC methods, was 0.934 and 0.939, respectively. The ensemble model, which combines the performance of the DenseNet121 architecture for both cropping methods, delivered outstanding results with an AUROC of 0.968, an accuracy of 0.938, a sensitivity of 0.980, and a specificity of 0.903. Furthermore, distinct trends observed in gradient-weighted class activation mapping images with the two cropping methods suggest that our deep learning model not only evaluates the lymph node itself, but also distinguishes subtler changes in lymph node margin and adjacent soft tissue, which often elude human interpretation. **Conclusions:** This research demonstrates the promising performance of a deep learning model in accurately detecting malignant ALNs in breast cancer patients using CT images. The integration of clinical considerations into image processing and the utilization of ensemble methods further improved diagnostic precision.

## 1. Introduction

Breast cancer stands as the most prevalent form of malignancy and a leading contributor to cancer-related fatalities among women worldwide [1]. In breast cancer, the axillary lymph node (ALN) is the most common site of metastasis. Accurately determining the presence of ALN metastasis is crucial for clinical staging, prognosis evaluation, and treatment planning for breast cancer patients [2].

To confirm ALN metastasis pathologically, sentinel lymph node dissection and ALN dissection are recommended. Although sentinel lymph node dissection is less invasive compared to ALN dissection, both procedures are invasive and can lead to lifelong complications, such as lymphedema and restricted shoulder movement. Therefore, non-invasive radiologic methods (ultrasound, magnetic resonance imaging [MRI], computed tomography [CT], positron emission tomography [PET]-CT) are recommended preoperatively. The clinical stage and treatment plan are determined based on the results of the radiologic examination.

CT is valuable due to its accessibility, ease of examination, and reproducibility. For these reasons, it is performed as a baseline examination for breast cancer patients and as a surveillance method to evaluate recurrence after treatment completion. Traditionally, the CT diagnostic criteria for metastatic lymph node (LN) include structural features such as size, shape, texture, margin, and enhancement patterns. Among these criteria, size, except for factors that may be influenced by a radiologist’s subjective decision, remains the most reliable factor. A short axis diameter > 1 cm is generally accepted as a threshold for malignancy [3,4]. However, the sensitivity of CT is often compromised by a high false-negative rate, as up to 67–74% of metastatic LNs have been reported to have a normal size of less than 1 cm [5,6]. 

In predicting ALN metastasis in breast cancer patients, CT demonstrates a sensitivity of 76–78% and a specificity of 75–97% [7,8]. The consistently observed low sensitivity may lead to the underestimation of the clinical stage, which can result in mistreatment. Therefore, supplementary diagnostic methods are required to enhance the accuracy of ALN detection by radiologic methods, including CT.

With the current advances in artificial intelligence (AI), specifically deep learning and machine learning, these technologies can enhance traditional diagnostic methods for ALN metastasis detection in breast cancer. Researchers have developed various AI models to detect LN metastasis of breast cancer using radiologic images, with several models—mainly employing MRI and ultrasonographic images—showing high performance, with area under the receiver operating characteristic curve (AUROC) values ranging from 0.71–0.99 [9].

In contrast to other radiologic methods, AI-based studies using CT scan to detect ALN metastasis in breast cancer are limited. To our knowledge, only two retrospective studies using CT scan have been conducted, with reported AUROC values of 0.817–0.969 [10,11]. Since studies using CT scan are very limited, it has not been clearly established which image preprocessing protocol or optimal convolutional neural network (CNN) model should be used for the best analysis results. Therefore, we conducted this study to develop a novel deep learning-based model to differentiate metastatic ALN in breast cancer patients from benign ALN based on CT scan and to validate the diagnostic performance of the system.

## 2. Materials and Methods

### 2.1. Study Subjects

A total of 1128 axial CT images of ALN, comprising 538 malignant and 590 benign lymph nodes, were collected. These images were obtained from 303 and 220 breast cancer patients, respectively, who underwent preoperative CT scans between January 2012 and July 2022 at Hallym University Medical Center. The characteristics of all malignant or benign LNs were pathologically confirmed by either percutaneous core needle biopsy or surgery. All images were obtained from contrast-enhanced chest CT scans, and non-enhanced CT/low-dose CT images were not included in this study. Patients who had received neoadjuvant treatment prior to their CT scan were excluded from the study. This retrospective study protocol was approved by the Ethics Committee of the Institutional Review Board at Hallym University Medical Center, Anyang-si, South Korea (IRB no. HALLYM 2023-07-017).

### 2.2. CT Imaging

All CT images were obtained using 64- to 128-channel multidetector CT scanners (SOMATOM Definition Flash, SOMATOM Definition Edge, Somatom Force, Siemens Medical Solutions, Erlangen, Germany; Brilliance, Philips Medical Systems, Eindhoven, The Netherlands). The tube voltage varied in the range of 80, 100, 120, or 140 kVp, and the current ranged from 45 to 714 mAs. The pitch factor was 0.6 or 1.2, and the detector collimation was 128 × 0.6 mm or 192 × 0.6 mm. The gantry rotation time ranged from 0.25 to 0.5 s. The pixel size ranged from 0.55 to 0.99 mm, and the slice thickness and spacing of axial images were 3 mm, respectively. Iterative reconstruction was applied using the ADMIRE version 2 reconstruction method (Siemens Healthineers, Erlangen, Germany).

### 2.3. Image Analysis Methods

**Figure 1:**
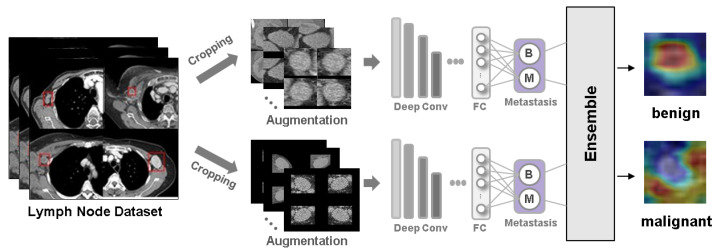
Overview of the CNN-based workflow.

In this study, we employed various CNN architectures, two different cropping methods, and an ensemble method to develop an optimal deep learning model that incorporates clinical implications for distinguishing metastatic ALN from benign ALN. Figure 1 illustrates the overall structure of the proposed methods. The steps include data collection, image conversion, cropping techniques, data augmentation, CNN model training, and the final integration using the ensemble method. Detailed information is described in the following subsections.

#### 2.3.1. Image Conversion

Acquired CT images are composed of Hounsfield Unit (HU) values and have a range of 4096 (12 bits). For deep learning-based analysis, the CT images are converted into grayscale images with a range of 256 (8 bits) by applying the same window level of 60 and window width of 40 as used in CT interpretation.

#### 2.3.2. Protocol for LN Bounding Box Generation

**Figure 2:**
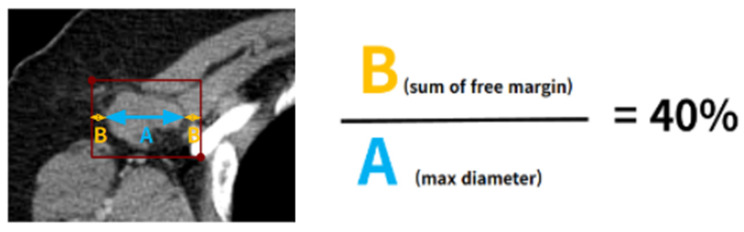
Illustration of Bounding box strategy: detailing margin and adjacent soft tissue around target lymph node.

The acquisition of ALN images was performed by following protocol. (i) Axial CT image which crossing center line of target LN; (ii) one axial CT image for one target LN; (iii) a maximum five target LNs from one patient. After acquiring axial CT images for the target LNs, a clinical expert manually annotated a region of interest (ROI) using the software “Labelme, version 5.4.0”. This ROI was delineated with a bounding box designed to encompass both the margin and the adjacent soft tissue of the LNs. The sum of margins was set to be 40% of the maximum diameter of the LN (as shown in Figure 2). 

#### 2.3.3. Crop Strategies for Image Analysis

**Figure 3:**
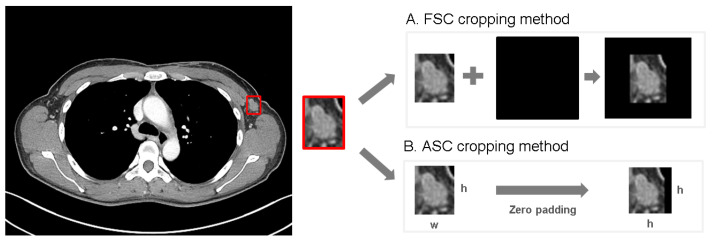
Two different cropping methods. (**A**) illustrates the fixed size crop method that reflects the actual size information, and (**B**) illustrates the adjustable square crop method that adjusts and equalizes the size information.

After obtaining the image of the target LN, it underwent processing using two distinct cropping methods prior to analysis with various CNN architectures (Figure 3). These cropping methods differed based on whether they incorporated the actual size information of the LN, which is a crucial criterion for distinguishing malignant LNs.

The first method is the fixed size crop (FSC) method, which reflects the actual size information of the LN. In this method, bounding box images are converted to their actual size in mm^2^ using the pixel spacing information from the DICOM header and aligned with the center point of a preset, fixed-size area. The fixed-size area is set at 55 mm square to encompass the majority of ALN images, with an additional 5 mm margin on each side. If a bounding box image exceeds the fixed-size area, it is resized to fit within that area. 

The second method is the adjustable square crop (ASC) method, which adjusts and equalizes the actual size information of the LN. In this method, bounding boxes are adjusted to form a square shape based on the longer side by applying zero padding. The longer side of the extracted bounding box is used as a reference for the square adjustment, while the shorter side is compensated using zero padding, resulting in a square shape. 

#### 2.3.4. CNN Architectures

Since different CNN architectures exhibit varying performance in image classification, this study aims to explore the unique features and effects of each architecture and evaluate their performance in accurately distinguishing between benign and malignant LNs in CT images. ResNet [12] introduces residual connections, a structure that directly adds the output from the preceding layer to the input of the current layer. This mitigates the vanishing gradient problem and effectively trains very deep neural networks. DenseNet [13] employs a densely connected structure where each layer is closely connected to the preceding layer, often referred to as “dense connections”. This design facilitates efficient gradient propagation and information reuse, leading to more efficient training and higher accuracy. EfficientNet [14] is designed to optimize network depth and width, achieving high performance with fewer parameters. It comes in various versions, such as B0 to B7, each suitable for different applications.

#### 2.3.5. Ensemble Method

**Figure 4:**
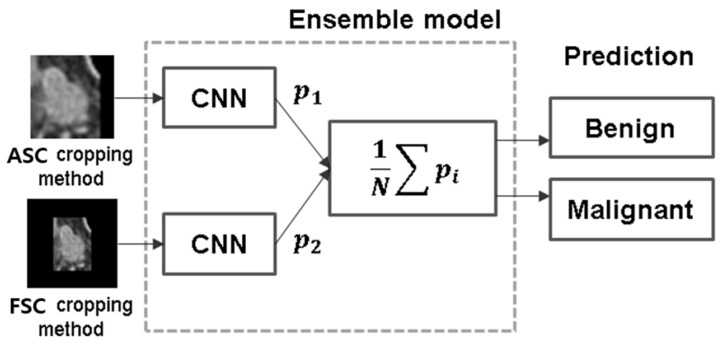
Ensemble method: selected top CNNs from FSC and ASC cropping methods for integrated performance.

The primary objective of our study is to accurately distinguish between malignant and benign LNs using CNN architecture. To achieve this goal, we applied ensemble methods to enhance overall performance, reduce prediction variance, and improve the final classification results [15,16]. This approach involves selecting the two best-performing CNN architectures from each cropping method (FSC and ASC methods) and combining them using the unweighted average ensemble method to generate the final results. Figure 4 provides an illustration of the ensemble method.

#### 2.3.6. Learning the Network 

The pretrained weights from the ImageNet dataset [17] were used to initialize the trainable parameters. The CNN classification models were trained using an augmented dataset that involved ±20° rotation and flipping to balance the class ratios, all on the same set of training samples. The training data were resized to 224 × 224, and the optimization of the training was performed using the Adam optimizer. The total number of epochs was set to 100, and the learning rate was set to range from 10^−3^ to 10^−5^. Two different strategies were employed for decreasing the learning rate: reducing it by a factor of 10 every 10 epochs and by a factor of 10 if no additional decrease was observed in the tuning dataset. The training process was conducted on a server equipped with an Intel(R) Xeon(R) Silver 4216 CPU @ 2.10 GHz, 256 GB RAM, and NVIDIA GeForce RTX 3090 (24 GB) in Ubuntu 20.04.1.

## 3. Results

This study included a total of 1127 LN images acquired from 523 patients. The entire dataset was randomly split into training, tuning, and test datasets in a ratio of 80%, 10%, and 10% three times independently. To ensure that ALN images of the same class from a single patient were not included simultaneously during the splitting process, the data were divided based on the patient identification number. The detailed composition of the first split is shown in Table 1. The training dataset consisted of 890 ALN images from 417 patients, while the tuning dataset included 113 ALN images from 53 patients. The test dataset consisted of 124 ALN images obtained from another 53 patients. 

### 3.1. CNN Performance Based on Cropping Methods

Table 2 and Table 3 present the results for each model using two different crop methods. Performance was evaluated using accuracy, AUROC, specificity, sensitivity, negative predictive value (NPV), positive predictive value (PPV), and F1 Score, with calculations performed at the threshold that maximizes Youden’s J statistic [18]. For each metric, the mean value and standard deviation of the results are calculated through independent training and evaluation using datasets created from three random splits. Furthermore, we selected the model that demonstrated the best performance and conducted a statistical comparison of its AUROC result with those of the other models using the DeLong test.

In the results with the ASC cropping method (Table 2), DenseNet121 demonstrates excellent performance in terms of AUROC, accuracy, sensitivity, and specificity, effectively classifying true positive and true negative cases. High PPV and NPV emphasize the accuracy of positive and negative predictions, ultimately achieving a high F1 score. Although EfficientNet B7 and ResNet152 also demonstrate good overall classification performance, EfficientNet B7 generally exhibits lower performance compared to DenseNet. ResNet152 shows relatively lower specificity, resulting in lower PPV, NPV, and F1 scores as well. To assess the statistical significance of these differences, we conducted the DeLong test using the same dataset that generally showed the best performance among the datasets created from three random splits. The DeLong tests were performed to compare the AUROC results between DenseNet121, which demonstrated the best performance, and ResNet152 as well as EfficientNet B7, resulting in *p*-values of 0.292 and 0.274, respectively. These outcomes indicate that there are no statistically significant differences in AUROC between DenseNet121 and the other models.

In the results with the FSC cropping method (Table 3), DenseNet121 also demonstrates great performance in AUROC, sensitivity, and NPV, effectively classifying both positive and negative cases. Furthermore, the F1 score reflects a balance between model accuracy and positive predictions, highlighting its exceptional classification performance. EfficientNet B7 shows good classification performance in certain aspects, but it exhibits relatively lower accuracy compared to DenseNet121 and ResNet152. ResNet152 excels in specificity and PPV, but shows relatively lower performance in sensitivity, NPV, and AUROC. As indicated by the DeLong test results, there were no significant differences in the AUROC results between DenseNet121 and the other models.

### 3.2. Performance of Ensemble Model

**Figure 5:**
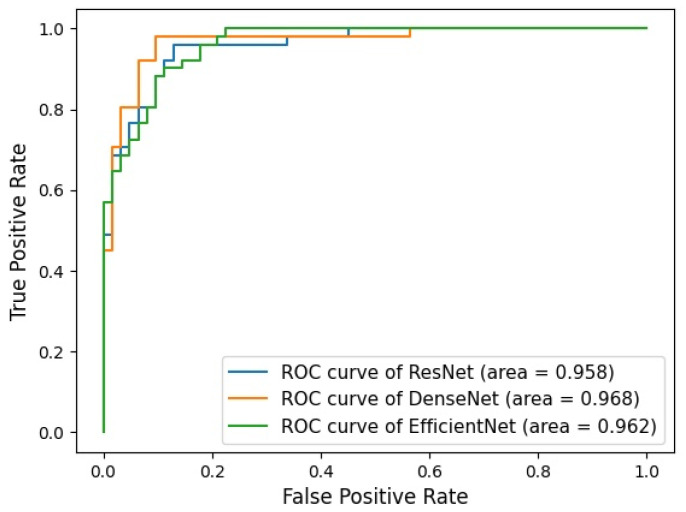
Receiver operating characteristic curve for three ensemble models classifying of benign and malignant ALN.

Table 4 presents the performance and 95% confidence intervals for three ensemble models based on each CNN architecture and Figure 5 shows the receiver operating characteristic (ROC) curves. For each cropping method, DenseNet121 consistently showed the best performance compared to other CNN architectures, and in the result of the ensemble model, DenseNet121 also exhibits the highest performance across all evaluation metrics. It achieves a sensitivity of 0.980 and specificity of 0.903, along with PPV and NPV of 0.893 and 0.982, respectively. The model demonstrates its effectiveness in classifying both positive and negative cases, as indicated by its F1 Score of 0.935, accuracy of 0.938, and AUROC of 0.968.

### 3.3. Gradient-Weighted Class Activation Mapping (Grad-CAM)

**Figure 6:**
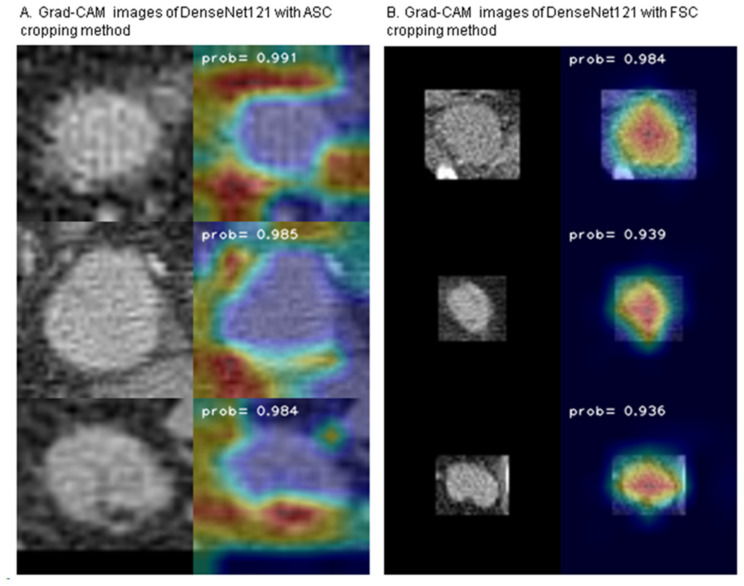
Representative images of Grad-CAM for malignant ALN using two different cropping methods with DenseNet121. (**A**) illustrates the ASC method, and (**B**) shows FSC method.

We utilized Grad-CAM to assess whether applying clinical implications to image processing affects the result of the deep learning-based analysis. Figure 6 shows representative images of original and overlaid Grad-CAM for malignant ALN using two different cropping methods (ASC and FSC methods) with DenseNet121. In the FSC method, which preserves actual size information, Grad-CAM for malignant ALN highlights the lymph nodes themselves. However, in the ASC method, which adjusts and equalizes size information, Grad-CAM tends to emphasize the margin and adjacent soft tissue of malignant lymph nodes.

## 4. Discussion

The primary aim of this study was to differentiate between malignant and benign ALN in axial CT images of breast cancer patients using CNN architectures. Our methodology incorporated a predefined protocol for image processing, different cropping methods based on clinical implications, architectural considerations, and ensemble methods. The study yielded promising results in detecting malignant LNs in terms of AUROC, accuracy, sensitivity, and specificity.

In our study, the ensemble model, which combines the performance of the DenseNet121 architecture for both FSC and ASC cropping methods, delivered outstanding results with an AUROC of 0.968 and an accuracy of 0.938. These results significantly outperformed the prediction accuracy of clinical experts. When compared to the prior best-performing AI model that used the DA-VGG19 model on 401 breast cancer patients (which reported an AUROC and accuracy of 0.969 and 0.909, respectively) [10], our results were comparable or slightly superior. To ensure the reliability of the analysis results, we trained and evaluated each CNN architecture with datasets created from three random splits and experimented with three CNN architectures separately. Additionally, clinical experts, including one radiologist and one medical oncologist, reviewed the Grad-CAM images to determine whether AI identified specific regions of clinical interest to distinguish malignant from benign LNs.

An important feature of our research, setting it apart from previous studies, is the incorporation of a unique predefined protocol during the early phase of image processing, specifically during bounding box generation. Rectangular bounding boxes were created according to a predefined protocol wherein the sum of the free margins was set at 40% of the LN’s maximum diameter. This protocol holds clinical significance, as it not only evaluates the LN itself, but also observes the changes in its margin and adjacent soft tissue in the event of metastasis.

During image processing, two distinct cropping methods were applied: the FSC method and the ASC method. Notably, a high level of AUROC and accuracy was also attained with ASC methods in which the size of the LNs—a criterion conventionally deemed pivotal by radiologists—was adjusted and equalized. This result suggests that AI’s strength lies not merely in evaluating size, but in discerning subtler features such as LN margin characteristics and adjacent soft tissue changes which often elude human interpretation. The trends observed in Grad-CAM images using ASC cropping methods support this hypothesis. Considering that metastasis is often confirmed after the biopsy of small (<1 cm) lymph nodes in actual clinical practice, this pivot towards a comprehensive nodal feature assessment may augment clinical diagnostic precision and address the false-negative issue.

The contrast in trends between Grad CAM images obtained using the FSC method and the ASC method is intriguing. The regions of interest identified with AI exhibit complementary features for each cropping method, and from a clinical perspective, these findings suggest that ensemble methods, which leverage the strengths of individual models while compensating for their weaknesses, could potentially yield a robust synergistic effect in the detection of malignant LNs. 

Recently, an AI-based approach using radiologic images to predict ALN metastasis in breast cancer patients is actively underway. The most common radiological methods used in radiomics for classifying ALNs are MRI and ultrasound. After the release of multiple promising results from MRI-based AI models (AUROC of 0.913–0.996) [19,20] and ultrasonography-based AI models (AUROC of 0.912–0.916) [21,22], the latest machine learning/deep learning approaches for predicting malignant LN incorporate a multi-modal analysis model that combines radiomics and clinicopathological features [23,24]. Together with other currently published studies, our findings and the image processing protocol used in our study can be applied to develop optimal multi-modal models that combine different radiomics to maximize diagnostic accuracy.

To our knowledge, this study analyzed the largest data set from the largest number of breast cancer patients compared to previous studies predicting ALN metastasis using CT scan and achieved the best performance to date. The promising results of this study imply that a predefined image processing protocol considering the clinical features of lymph node metastases may have influenced the performance of the AI-based diagnostic model. Results using the ASC method also suggest that our model may have the ability to distinguish subtle radiological features that are difficult for humans to recognize. 

A limitation of this study is that there is a need for external validation on diverse datasets to ensure the model’s robustness and generalizability. Furthermore, to create a fully automated model that can be used in actual clinical practice as a clinical decision support model, an additional system that automatically detects ALN node location in CT scan should be developed.

## 5. Conclusions

In conclusion, this study successfully demonstrates the potential role of CNN architectures in improving the accuracy of detecting malignant ALN using CT images in breast cancer patients. The combination of tailored image preprocessing, architecture selection, and ensemble techniques has the potential to advance AI-assisted medical diagnostics, offering timely and precise treatment to breast cancer patients.

## Figures and Tables

**Table 1:** Detailed composition of the training, tuning, and test datasets in the initial dataset split.

	Whole Dataset		Training Set		Tuning Dataset		Test Dataset	
	Image N	Patient N	Image N	Patient N	Image N	Patient N	Image N	Patient N
Overall	1127	523	890	417	113	53	124	53
Malignant	538	303	422	241	53	31	63	31
Benign	589	220	468	176	60	22	61	22

**Table 2:** Performance of three CNN architectures using ASC method with three independent random splits.

	Accuracy	AUROC	Sensitivity	Specificity	PPV	NPV	F1 Score	*p*-Value *
ResNet 152 [12]	0.83 ± 0.039	0.929 ± 0.021	0.874 ± 0.068	0.878 ± 0.024	0.868 ± 0.02	0.885 ± 0.062	0.869 ± 0.028	0.292
DenseNet 121 [13]	**0.87 ± 0.043**	**0.939 ± 0.026**	**0.900 ± 0.043**	0.883 ± 0.037	**0.878 ± 0.033**	**0.904 ± 0.045**	**0.889 ± 0.038**	
EfficientNet B7 [14]	0.862 ± 0.019	0.927 ± 0.020	0.874 ± 0.075	**0.884 ± 0.052**	0.876 ± 0.052	0.888 ± 0.064	0.87 ± 0.013	0.274

The results include the mean values and their corresponding standard deviations. Bold represents the best performance in each metric. * Comparing AUROC result of DenseNet 121 with ResNet and EfficientNet using a DeLong test.

**Table 3:** Performance of three CNN architectures using FSC method with three independent random splits.

	Accuracy	AUROC	Sensitivity	Specificity	PPV	NPV	F1 Score	*p*-Value *
ResNet 152 [12]	0.851 ± 0.024	0.929 ± 0.023	0.858 ± 0.024	**0.9 ± 0.041**	**0.891 ± 0.034**	0.872 ± 0.033	0.874 ± 0.025	0.171
DenseNet 121 [13]	**0.875 ± 0.038**	**0.934 ± 0.03**	**0.921 ± 0.059**	0.844 ± 0.042	0.847 ± 0.03	**0.921 ± 0.063**	**0.881 ± 0.034**	
EfficientNet B7 [14]	0.814 ± 0.038	0.933 ± 0.024	0.893 ± 0.034	0.857 ± 0.039	0.853 ± 0.025	0.896 ± 0.037	0.872 ± 0.021	0.118

The results include the mean values and their corresponding standard deviations. Bold represents the best performance in each metric. * Comparing AUROC result of DenseNet 121 with ResNet and EfficientNet using a DeLong test.

**Table 4:** Performance for the three ensemble models based on different CNN models using two crop methods.

	Accuracy	AUROC	Sensitivity	Specificity	PPV	NPV	F1 Score
ResNet 152	0.912 (0.859–0.964)	0.958 (0.952–0.960)	0.961 (0.868–0.988)	0.871 (0.765–0.933)	0.860 (0.746–0.927)	0.964 (0.879–0.989)	0.907 (0.9–0.914)
DenseNet 121	**0.938** **(0.894–0.982)**	**0.968** **(0.965–0.971)**	**0.980** **(0.897–0.995)**	**0.903** **(0.804–0.954)**	**0.893** **(0.785–0.949)**	**0.982** **(0.908–0.996)**	**0.935** **(0.930–0.940)**
EfficientNet B7	0.894 (0.837–0.951)	0.962 (0.960–0.966)	0.902 (0.790–0.956)	0.887 (0.784–0.944)	0.868 (0.751–0.934)	0.917 (0.819–0.963)	0.885 (0.879–0.892)

The results include 95% confidence intervals. Bold represents the best performance in each metric.

## Data Availability

Data are available on reasonable request from the corresponding author. The underlying code for this study is available in [GitHub] and can be accessed via this link [https://github.com/pak14kr/LymphNode, accessed on 7 March 2024].
